# Effects of Early Weight Gain Velocity, Diet Quality, and Snack Food Access on Toddler Weight Status at 1.5 Years: Follow-Up of a Randomized Controlled Infant Formula Trial

**DOI:** 10.3390/nu13113946

**Published:** 2021-11-04

**Authors:** Julie A. Mennella, Alissa D. Smethers, Jessica E. Decker, Michelle T. Delahanty, Virginia A. Stallings, Jillian C. Trabulsi

**Affiliations:** 1 Monell Chemical Senses Center, Philadelphia, PA 19104, USA; asmethers@monell.org; 2 Department of Behavioral Health and Nutrition, University of Delaware, Newark, DE 19173, USA; jeosso@udel.edu (J.E.D.); mdelahan@udel.edu (M.T.D.); trabulsi@udel.edu (J.C.T.); 3 Children’s Hospital of Philadelphia, Philadelphia, PA 19104, USA; STALLINGSV@chop.edu

**Keywords:** rapid weight gain, infant formula, dietary intake, added sugar, snack foods, parenting, toddlers, snacks

## Abstract

This study followed children who participated in a feeding trial in which the type of randomized infant formula fed from 2 weeks significantly affected weight gain velocity during the first 4 months and weight-for-length Z (WLZ) scores up to 11.5 months. We focused on measures of anthropometry, dietary intakes, and parenting related to the provision of snack foods that were collected at the end of the trial (1 year) and the 1.5 years follow-up visit. We not only describe what toddlers are eating, but we also determined the independent and/or interactive effects of randomized formula group, early weight gain velocity, the nutrient content of the post-formula diet, and maternal snack food practices, on toddlers’ weight status. Diet quality underwent drastic changes during this 6-month period. As infant formula disappeared from the diet, fruit and 100% fruit juice intake increased slightly, while intake of “What We Eat in America” food categories sweetened beverages and snacks and sweets more than doubled. Added sugars accounted for 5% of energy needs at 1 year and 9% at 1.5 years. Generalized linear mixed models revealed that, independent of the randomized formula group, greater velocities of weight gain during early infancy and lower access to snacks as toddlers predicted higher WLZ and a greater proportion of toddlers with overweight at 1.5 years. Energy and added sugar intake had no significant effects. These findings add to the growing body of evidence that unhealthy dietary habits are formed even before formula weaning and that, along with improving early diet, transient rapid weight gain and parental feeding practices are modifiable determinants that may reduce risks for obesity.

## 1. Introduction

The evidence that infants’ early nutritional environment plays a crucial role in optimizing growth [1], developing a palate for a healthful diet [2], and reducing disease risks [3,4] provides additional importance for recent dietary guidance for children 2 years of age and younger [5,6,7,8]. After birth, the infant’s diet is unique, typically consisting of a sole source of liquid nutrition—human milk, human milk substitutes (infant formula), or both. Although infants fed formula gain weight faster during the first year [9] and are at greater risk for later obesity than breastfed infants [10,11,12], formula-fed infants are not a homogeneous group. Randomized controlled trials (RCTs) revealed that isocaloric formulas of different macronutrient composition had different effects on early rapid weight gain [13,14,15], a consistent and established risk factor for later obesity and other comorbidities [16,17,18].

In our RCT on healthy term infants, those randomized to be fed cow milk formula (CMF), the most commonly consumed type of infant formula [19], experienced accelerated weight gain during the first 4 months compared to the normative weight gain pattern of infants fed an extensive protein hydrolysate formula (EHF), due to both energy loss and intake mechanisms [13]. While within the range of typically growing healthy infants, the formula-induced differences in weight-for-length Z (WLZ) scores remained significantly different between the groups up to 11.5 months [13]—a time when children begin the transition to a more complex diet.

The children in this trial, the majority of whom were Black Americans, were phenotyped from 2 weeks to 1.5 years [13], from the time the randomized formula was the sole source of their nutrition to when formula was no longer a part of their diet. In the present study, we focused on these children when they were young toddlers, aged 1 year (i.e., last RCT visit) and 1.5 years (i.e., follow-up visit), and report on the food and nutrient contents of their diets and their mothers’ feeding practices specifically related to the provision of snack foods [20], an important context that can have either positive or negative consequences on their nutritional intake and weight status [21,22]. In addition to describing the diets and feeding practices of this contemporary cohort of dyads, we have the opportunity to investigate predictors of weight status because these children participated in a clinical trial in which the experimental treatment—CMF or EHF—had significant effects on velocities of early weight, an established a reliable predictor of risks for subsequent obesity [23]. Thus, the overarching goal of this follow-up study was to determine whether the randomized formula group (CMF, EHF), early weight gain velocity, the nutrient content of their diet, and their mothers’ feeding practices specific to snack foods had independent and/or interactive effects on toddlers’ weight status at the ages of 1 and 1.5 years.

## 2. Materials and Methods

### 2.1. Participants

Data were collected during a double-blind longitudinal RCT that investigated the effect of infant formula type on primary outcomes of growth and energy balance [13]. The study was conducted according to the guidelines of the Declaration of Helsinki, approved by the Office of Regulatory Affairs at the University of Pennsylvania Institutional Review Board (protocol number 815181), and registered online at clinicaltrials.gov prior to the start of the trial (NCT01700205). The study design, inclusion and exclusion criteria, and the CONSORT Table for the 1-year RCT were published previously [13]. Throughout the trial, no instructions were given to mothers on how or when to feed infant formula, solid foods, or beverages.

In brief, a racially diverse group of term infants, whose mothers decided to exclusively formula feed, were randomized to be fed either CMF (*n* = 59) or EHF (*n* = 54) formulas that were isocaloric but differed in composition (i.e., protein form and concentration, carbohydrate type). The intent-to-treat (ITT) RCT cohort of 113 infants was diverse in race/ethnicity (62% Black, 22% White, 16% other/more than one race), education status, and household income [13], all of which reflected the urban setting in which they lived: the city of Philadelphia [24]. As reported previously [13], there were no significant differences between the randomized groups in any measured characteristics of mothers or their infants at enrollment. Throughout the trial, the treatment groups did not differ in linear growth, and length-for-age Z scores increased in both groups at rates typical of healthy infants [13].

**Figure A1:**
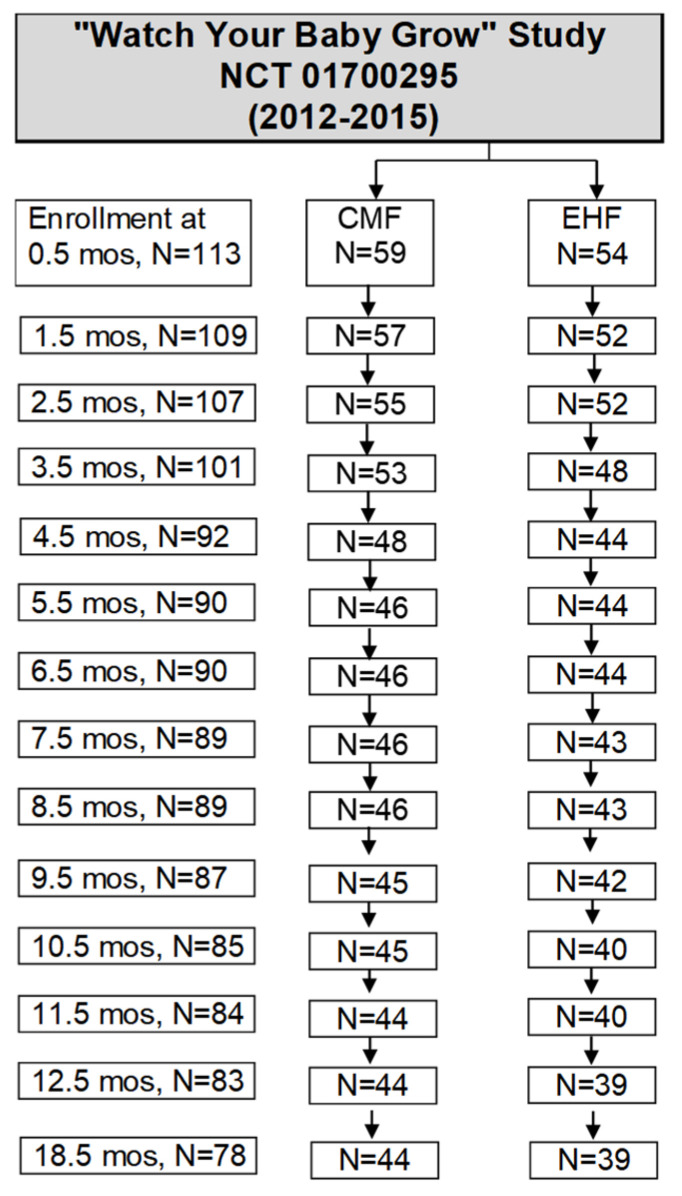
Trial profile of parent randomized controlled trial, NCT01700295. Number of dyads in two randomized groups that differed by type of formula fed (CMF, cow milk formula; EHF; extensive protein hydrolysate formula). Number (N) enrolled at each month of trial up to 12.5 months (1 year), and then 18.5-month (1.5 years) follow-up visit.

As shown in Figure A1, there were no differences between the groups in dropout rates and the completion rate for the 1-year trial was 73%. Most dropouts occurring during the first few months of the trial, primarily because mothers no longer wanted or were unable to participate. The focus of the present analysis was the 83 children who completed the trial at 12.5 months (referred to as 1 year), of whom 78 completed the follow-up visit at 18.5 months (referred to as 1.5 years), referred to herein as toddlers. In addition to the randomized formula group and individual differences in velocity of weight gain during early infancy (0.5 to 4.5 months), the following outcomes analyzed in the present study were collected during the 1-year and/or 1.5-years visits: (a) dietary intake; (b) maternal influences on snack food feeding, as determined by the Toddler Snack Food Feeding Questionnaire (TSFFQ) [20]; and (c) WLZ scores, from which we determined the proportion of children with overweight at 1.5 years.

### 2.2. Anthropometry

During each on-site visit at the Monell Center, children were weighed and measured in triplicate by research personnel certified in standard anthropometric techniques and with calibrated pediatric scales (Scale–Tronix, White Plains, NY, USA) and stadiometers (Harpenden Infantometer 702; Crymych, Dyfed, UK) that were accurate to 0.001 kg and 0.1 cm, respectively. Anthropometric data were converted to weight-for-length Z (WLZ) scores using World Health Organization growth standards, which were established using the breastfed infant as the normative model for growth [25], and to velocities of early weight (g/day) and length (cm/day) accretion, calculated by dividing the change of weight in grams or length in centimeters by the change of age in days [26] from 0.5 to 4.5 months (referred to as early weight gain velocity), from 4.5 months to 1 year, and from 1 year to 1.5 years. The weight status categories at 1.5 years were based on age- and sex-specific WLZ percentiles, with overweight was defined as WLZs greater than the 85th percentile (WLZ > 1.0364) [27].

### 2.3. Toddler Snack Food Feeding Questionnaire (TSFFQ)

When their children were 1 and 1.5 years, mothers completed the validated TSFFQ [20] that measures five meaningful and interpretable constructs related to caregiver’s influences on their children’s snack food intake and perceptions of their children’s attraction to these foods: the extent to which parents (a) allow children access to snacks foods (Allow Access); (b) have rules about snacking (Rules); (c) are self-efficient in managing snack foods (Self-Efficacy); and (d) are flexible in managing what, how much, and when sweets and snack foods are given to the child (Flexibility); and (e) how attracted their child is to sweets and snack foods (Child Attraction). Prior to administration of the questionnaire, mothers were shown a collage of popular manufactured high-energy snacks. Mothers scored each item using 5-point rating scales, with responses varying from never (1) to always (5); unaware of all (1) to aware of all (5); none of these (1) to all of these (5); or never (1) to at least once per day (5). Each construct was computed by averaging the item scores. Lower numbers represent less of a construct. The TSFFQ was completed by all but two mothers at 1 year (*n* = 81) and all mothers at 1.5 years (*n* = 78). Cronbach’s alpha, an estimate internal consistency and reliability for each factor, ranged from 0.76 to 0.92 at 1 year, and from 0.78 to 0.92 at 1.5 years. Ratings scores for each construct were averaged across the two time points; if one was missing, the score from the one time point was used.

### 2.4. Dietary Intake

Because infant formula remained the predominant source of nutrition until 10.5 months [13], we focused herein on the two sets of 3-day diet records obtained at the 1 year and 1.5 years on-site study visits. When children were 1 year, energy intake of formula was determined by a consecutive 3-day weighed bottle intake [13]; we standardized the number and size of the bottles since it can affect both formula intake and weight gain [28]. During the 3 consecutive days preceding the 1 year and 1.5 years on-site visits, mothers also prospectively recorded the types and amounts of all other liquids or foods ingested by their children for each 24-h period. Upon return, registered dietitians and research staff reviewed records.

We analyzed diet records using the Nutrient Data System for Research (University of Minnesota, Minneapolis, MN, USA) to determine daily intake of total energy, added sugar, and sodium. Dietary data were available for 67 of the 83 (81%) children at 1 year, and 73 of the 78 (94%) children at 1.5 years. For the remaining days, diet records either were not returned or were incomplete (e.g., no information on portion sizes, incomplete 24-h records, leaked formula bottles). At 1 year, we had 3 days of consecutive dietary data for 60 children, 2 days for 4 children, and 1 day for 3 children, and at 1.5 years, we had 3 days of dietary data for 53 children, 2 days for 8 children and 1 day for 12 children. Thus, the majority of records were for 3 consecutive days (1 year, 90%; 1.5 years, 73%). To determine whether energy intake differed based on the number of days of records, we compared the intake of all children (regardless of the number of days of diet records) with intake of children who had 3-days of diet records. The differences in mean energy intakes were negligible and not significant: 5 kcal/day at 1 year (*p* = 0.93) and 13 kcal/day at 1.5 years (*p* = 0.86).

We focused on intake of six of the mutually exclusive “What We Eat in America” (WWEIA) categories [29]: infant formula, snacks and sweets (e.g., savory snacks, crackers, sweet bakery products), sweetened beverages (e.g., soft drinks, fruit drinks), sugars (i.e., sugars, honey, sugar substitutes, jams, syrups, toppings), fruit, and 100% fruit juice. Because most US children have had some exposure to added sugar by the age of 2 years [30], despite the most recent dietary guidelines that recommend no added sugar intake in the diet of children younger than 2 years [7], we focused on intakes of categories of foods and beverages that contain added sugars as well as those that are nutrient-dense and contain natural sugars such as fruit to explore whether the children’s diet changed during this 6-month period. For the present analysis, the snacks and sweets category and the fruit category included commercial baby-food versions of these foods. For both ages, we determined daily energy (kcal), added sugar (g), and sodium (mg) content of the total diet and the six WWEIA categories, as well as the percentage of children consuming each food category at a given age. Energy (kcal/day) from added sugars, defined by the Food and Drug Administration as sugars, syrups, and concentrated fruit or vegetable juices added to foods during processing or preparation, does not include sugars found naturally in foods [31]. Because the labeling requirement for added sugars does not apply to infant formula, it was not included in the determination of daily added sugar intake [32]. Caloric intake of added sugars was based on 4 kcal per gram. Dietary data were analyzed as kcal/day or percent of daily energy.

### 2.5. Statistical Analyses

Statistical analyses and hypothesis testing progressed in a procedural manner from testing for normality, to exploratory data analysis for identifying potential covariates, to modeling dependent outcomes. We conducted analyses on the subsample of children who completed the trial (*n* = 83) to ensure that the two groups did not differ in demographic characteristics (e.g., race/ethnicity, maternal education, household income) or other baseline characteristics (e.g., birth weight, infant sex, maternal age, maternal body mass index) that could impact later weight status [33,34,35] and to ensure that the groups did differ in velocities of early weight gain, as reported for the ITT analysis of the 113 infants [13]. Variables were tested for normality using the Shapiro–Wilk test. Bivariate relationships between WLZ at 1.5 years and baseline characteristics, anthropometry, diet outcomes, and TSFFQ scores were examined using t-tests for group comparisons and Pearson correlations for continuous associations. Variables that were significant in the bivariate relationships were then included in General Linear-Mixed Effects models to examine predictors of the WLZ outcomes. A random intercept for infant and random slope for time were used to allow trajectories to vary across subjects. Fixed effects in the model included randomized infant formula treatment group, using the CMF group as the reference group, and time (1 year, 1.5 years), which was treated as a continuous variable. 

After comparison of models using potential correlation matrices, an unstructured correlation matrix was chosen for the correlations with WLZ scores at 1 year and 1.5 years for each toddler. Significant effects were followed by splitting continuous variables into meaningful groups to illustrate findings and conduct further analyses. Data were analyzed using Stata/IC version 17.0 (Statacorp LLC, College Station, TX, USA) and Statistica version 14.2 (StatSoft Inc., Tulsa, OK, USA), with a significance criterion set at *p* < 0.05, and are expressed as *n* (percent [%]) or mean (standard error of mean [SEM]). 

## 3. Results

### 3.1. Participant Characteristics

We first established that of the 83 infants who remained in the trial at 1 year, there were no significant differences between the groups at enrollment. As shown in Table 1, there were no significant group differences in any measured characteristics of the infants or their mothers at baseline, as was previously observed for the entire cohort [13].

Next, we established that the type of infant formula fed had significant effects on early weight gain velocity but not length gain velocity, as previously reported [13,36]. Of the 83 infants who completed the RCT at 1 year, the CMF group gained 29.2 g/day (95% CI: 27.6–30.8), and the EHF group gained 26.1 g/day (95% CI: 23.9–28.4) from 0.5 to 4.5 months (Table 2). From 4.5 months to 1 year, weight gain velocities and the patterning of weight curves did not differ between the formula treatment groups, although until 11.5 months WLZ scores were significantly higher for the CMF group than for the EHF group [13]. At 1 year and at 1.5 years, the groups no longer differed in WLZ scores (Table 2), and from 1 year to 1.5 years, groups did not significantly differ in velocities of weight gain (CMF vs. EHF: 7.0 ± 0.5 vs. 7.5 ± 0.5 g/day; *p* = 0.51) or length gain (CMF vs. EHF: 0.033 ± 0.001 vs. 0.033 ± 0.001 cm/day; *p* = 0.82). The two groups did not differ in rates of linear growth. Neither did the two randomized groups differ in the age of introduction to solid foods (CMF vs. EHF: 5.4 ± 0.3 vs. 5.1 ± 0.3 months; *p* = 0.43); maternal TSFFQ ratings (*p* = 0.31); or total energy intake at 1 year (*p* = 0.99) and 1.5 years (*p* = 0.25). 

### 3.2. Dietary Changes from 1 Year to 1.5 Years

**Figure 1:**
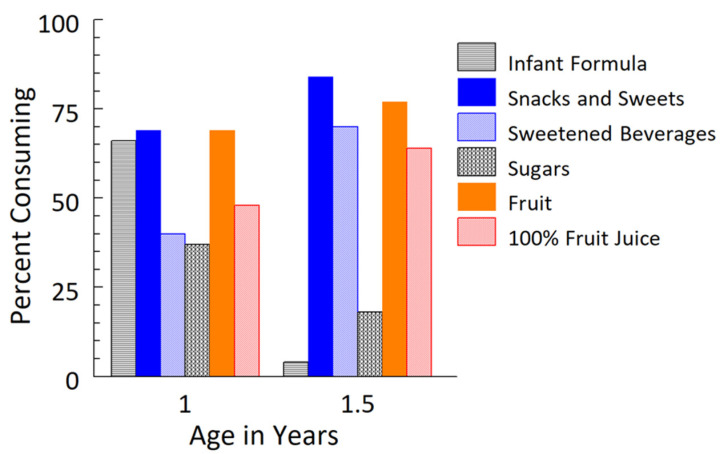
Percentages consuming selected What We Eat in America (WWEIA) food categories at 1 year and then at 1.5 years. Diet records available for *n* = 67 of 83 toddlers at 1 year and *n* = 73 of 78 toddlers at 1.5 years. Diet records not returned or incomplete for remaining. Snacks and sweets category included baby-food snacks and sweets, and fruit category included baby-food fruit.

The percentages of children consuming each WWEIA food category at both ages are shown in Figure 1, and the mean daily intake and percent daily energy by food type and category are summarized in Table 3. The percentage of children fed any infant formula decreased from 66% to 4% from 1 year to 1.5 years, with formula accounting for only 1% of the daily energy at 1.5 years. During this 6-month period, added sugar intake doubled and accounted for, on average, 5% of caloric intake (range: 1–24%) at 1 year, and 9% (range: 1–23%) at 1.5 years.

The majority of 1-year-old and 1.5-years-old toddlers were ingesting food from the snacks and sweets and fruit categories at both ages. The percentage of children consuming sweetened beverages on a given day increased from 40% at 1 year to 70% at 1.5 years, and for 100% fruit juice (e.g., apple, orange, white grape), from 48% to 68%. Snacks and sweets commonly consisted of savory or sweet puffs, chips, crackers, biscuits, cookies, and frozen desserts, foods typically high in added sugars or sodium [37,38], and were fed to 69% and 84% of the children at 1 year and 1.5 years, respectively. The contribution of snacks and sweets to children’s daily energy intake increased from 5% at 1 year to 12% at 1.5 years, and of sweetened beverages, from 2% to 5%. Some mothers used sugars to sweeten the foods or liquids fed to their children, but this contributed to less than 10 kcal/d and did not change during this 6-month observation period. When combined, the WWEIA categories of snacks and sweets, sweetened beverages, and sugars accounted for 8% (±1) and 17% (±1) of the daily energy intake at 1 year and 1.5 years, respectively.

At both ages, children were less likely to be fed commercial baby-food snacks and sweets, and baby-food fruit, than the nonbaby foods versions of these foods. Baby-food fruit contributed to 29% of the caloric intake of fruit at 1 year and only 5% at 1.5 years. Likewise, baby-food snacks and sweets contributed to 40% of the energy intake of this food category at 1 year, decreasing to 9% at 1.5 years. Fruit accounted for less of toddlers’ energy intake than snacks and sweets at 1.5 years (Table 3). The greater the energy from fruit and fruit juices combined, the less energy from snacks, sweetened beverages, and sweets at 1.5 years (*p* = 0.009). The more added sugar in the diet, the more sodium it contained (*p*’s < 0.001).

### 3.3. The General Linear Mixed Effects Model: Independent Effects of Early Weight Gain Velocity and Parenting Specific to Snack Foods

Bivariate correlations revealed that weight gain velocity from 0.5 to 4.5 months (*p* < 0.000) and percent daily energy from fruit (*p* = 0.03) were positively correlated, and TSFFQ Allow Access ratings were negatively (*p* < 0.001) correlated, with WLZ at 1.5 years, and thus, were included in the model. However, when tested, percent energy from fruit did not remain significant (*p* = 0.15) and was removed. No other dyad characteristic (Table 1 and Table 2), TSFFQ construct, or diet outcome (Table 3) correlated with WLZ scores.

Table 4 summarizes the findings from the model and the *p* values for independent and interactive effects. We found no significant effects of the randomized infant formula treatment group, but both early weight gain velocity and maternal Allow Access ratings were significant predictors of toddlers’ WLZ scores that were each independent of formula treatment group. Faster gains in weight during the first 4 months predicted higher WLZ scores, whereas higher Allow Access ratings predicted lower WLZ scores, among toddlers.

To further explore these significant predictors from the model, we stratified the infants’ early weight gain velocities (0.5–4.5 months) and the maternal Allow Access ratings at 1 and 1.5 years by sample-specific percentiles (<25th, 25th–75th, >75th). We then conducted separate general linear models to determine whether these percentile groups were associated with differences in (a) body weight status (WLZ scores) at 1.5 years and (b) the proportion of toddlers with overweight, defined as WLZ values greater than the 85th percentile (WLZ > 1.0364), at 1.5 years [28].

**Figure 2:**
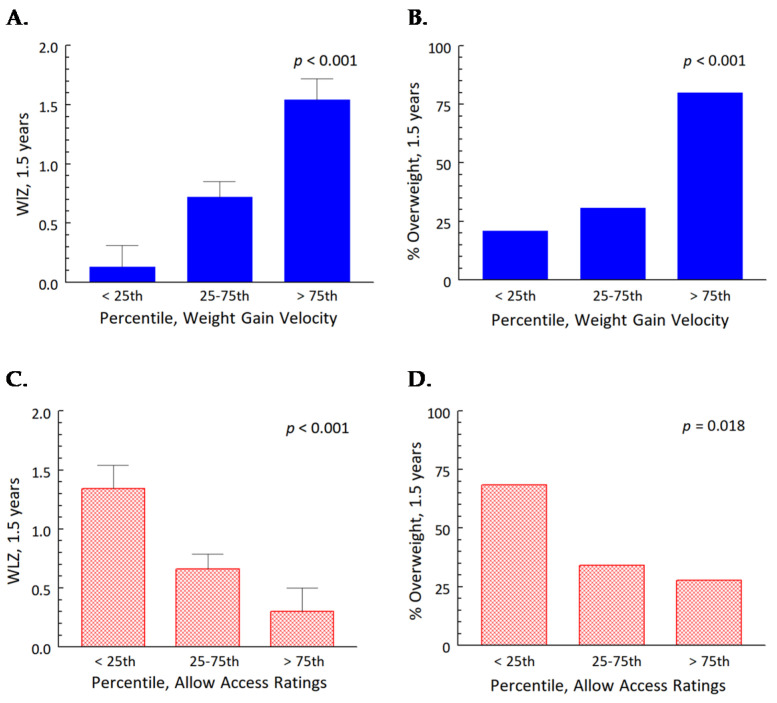
WLZ scores of toddlers and percent overweight at 1.5 years (*n* = 78) by percentile category of early weight gain velocity (g/day) from 0.5 to 4.5 months (blue bars) and maternal Allow Access ratings at 1 and 1.5 years (red hatched bars). Higher velocities in weight gain (>75th percentiles) were associated with higher WLZ scores (**A**) and a greater proportion of toddlers with overweight at 1.5 years, (**B**) whereas higher ratings by mothers for allowing their toddlers access to snacks and sweets were associated with lower WLZ scores (**C**) and a smaller proportion with overweight at 1.5 years (**D**).

For early weight gain velocity, the >75th percentile group gained >30.7 g/day from 0.5 to 4.5 months; the 25th–75th percentile group, 23.2–30.7 g/day; and the < 25th percentile group, <23.2 g/day. We found significant effects of percentile group for early weight gain velocity: toddlers in the >75th percentile for early weight gain velocity had higher WLZ scores at 1.5 years (*p* < 0.001; Figure 2A), and a greater proportion were classified as overweight at 1.5 years (*p* < 0.001; Figure 2B). 

For TSFFQ Allow Access ratings, the >75th percentile group scored >3.15; the 25th–75th percentile group, 2.30–3.15; and the <25th percentile group, <2.30. We found a significant effect of percentile group for Allow Access ratings: mothers in >75th percentile group had toddlers with significantly lower WLZ scores at both ages (*p* < 0.001; Figure 2C), a smaller proportion of whom were overweight at 1.5 years (*p* = 0.018, Figure 2D).

## 4. Discussion

How quickly children gained weight during the first 4 months of formula feeding, and how much their mothers allowed them access to snack foods and sweets at 1 and 1.5 years, predicted the weight status of this contemporary cohort of mother-child dyads who represent the cultural and socioeconomic diversity of the city in which they live: Philadelphia [24]. These predictions, however, were in opposite directions: faster velocities of weight gain during the first 4 months, when more than 95% of their energy intake was from infant formula [13], predicted higher WLZ scores at 1 and 1.5 years, and a greater proportion of toddlers who had been in the greater than 75th percentile for early weight gain (0.5 to 4 months) were overweight at 1.5 years. In contrast, mothers who allowed more access to snacks and sweets had toddlers with lower WLZ scores at 1.5 years, and a smaller percentage of children in the >75th percentile for Allow Access ratings were overweight at 1.5 years. There were no significant effects on the weight status of the toddlers from the caloric intake from any of the food categories, including the WWEIA categories of snacks and sweets or fruit, or the energy from added sugar.

In the ITT sample of 113 children in the parent RCT [13], as well as this subsample of 83 children who completed the trial, the randomized treatment (type of infant formula fed during the first year) had significant effects on early weight gain velocity and WLZ. Beginning within 1 month after randomization (when infants were 1.5 months old) and persisting until 11.5 months, the CMF group had higher WLZ scores than the EHF group [13]. By the age of 1 year (final visit of the RCT) and then at the 1.5 years follow-up visit, there were no longer differences in WLZ scores between the two groups. Yet the consequence of the experimental treatment—velocities of weight gain during the first 4 months—predicted the weight status of these infants at 1.5 years. This finding extends prior report that a greater proportion of children with early rapid weight gain were overweight when 10.5 months of age, 8 months earlier than 1.5 years [36]. Unlike retrospective and prospective observational studies, which cannot elucidate causal relationships, the RCT design of the parent study and the balance between the groups for a variety of maternal and infant characteristics that are known confounders for later weight status [33,34,35] minimized selection biases and enabled us to determine the effects of infant formula diet, early weight gain velocity, and other significant covariates in predicting body weight status of toddlers, while keeping other variables constant.

The finding that toddlers whose mothers allowed them more access to sweets and snacks were less likely to be overweight is consistent with prior research that parental restriction, not access, predicts higher WLZ scores [21,39]. The Allow Access construct of the TSFFQ focuses on the decisions mothers make in structuring their child’s food environment and the extent to which they give them access to sweets and snacks [20]. Items include questions on how often snacks are given, including special occasions; whether the toddler is aware of, asks for, or has access to snacks; and whether the home is free of snacks or not. As suggested by Corsini et al. [22], this Allow Access construct may be a proxy for variability in the mothers’ role as nutritional gatekeepers and may indicate positive aspects of (a) the control they have over their toddler, (b) the accessibility of extra foods in the home environment, and (c) parenting style regarding all foods. However, unlike early weight gain velocity that occurred from 0.5 to 4.5 months, the TSFFQ was completed by mothers during the same visits that the anthropometric measures of their children were made (i.e., 1 year, 1.5 years). While the TSFFQ could not be completed earlier since it is specific to feeding toddlers [20], the amount of access to snack foods that mothers gave their children may be in response to their child’s body weight status, not the cause [40].

The energy and added sugar content of the diet overall or from snacks and sweets, sweetened beverages, or fruit did not predict body weight status of the toddlers. We have several, not mutually exclusive, explanations for this finding. Firstly, some mothers may not be accurate in their records, since random error can occur due to within-child variability in energy intake from day to day or due to mothers forgetting to record a snack or beverage or making portion size errors [41]. One study found that mothers of toddlers overestimated energy intake because of errors in estimating portion size compared to that of 3-day weighed records [42]. Although we determined the energy intake of formula by weighed bottle intakes, and mothers recorded what their child ate in real time on 3 consecutive days and quantified amounts in household units (e.g., cups, fluid ounces), we did not have weighed records for the foods and beverages. However, by the time these mothers reached the 1.5-years follow-up visit, they had recorded a total of 17 days of diet records for their children, each reviewed by registered dietitians or research staff. Thus, we are confident in the integrity of the records. Alternatively, how mothers conceptualized a food, particularly when completing the 3-day record, possibly led to underreporting of some foods by some and, in turn, inconsistencies with their TSFFQ ratings. Research by Fisher and colleagues [43,44] on low-income mothers of toddlers living in Philadelphia found that some mothers considered snack foods, particularly those that are sweet tasting, to be “junk food” and consequently not “real food”. Whether these mothers would be less likely to record such foods on the diet records is not known.

Secondly, how WWEIA defines “snacks and sweets” may not be how mothers define them [45,46], particularly when completing the TSFFQ. Mothers may define snacks in different ways: as a particular food, as foods that require less preparation than meals, as foods fed during certain times of the day or in certain locations, or as foods used to reward or punish their children [21,43,46,47,48,49]. The WWEIA categorization of snacks and sweet includes items such as savory snacks, crackers, meal bars, sweet bakery products, and candy, and it does not include nutrient-dense foods such as fruit or vegetables, which are viewed by some mothers as snacks. Such discrepancies would weaken the association between dietary intake and parenting around snacks.

Thirdly, the quality of children’s diet underwent drastic changes from 1 year to 1.5 years. While the percentage of energy from infant formula decreased to negligible amounts by 1.5 years, added sugar doubled during this 6-month period. The prevalence of toddlers eating snacks and drinking sweetened beverages and the nontrivial impact these foods have on daily energy, added sugar, and sodium intake are consistent with prior research [37,50,51,52,53,54,55]. However, we do not know when during this 6-month period these dietary changes occurred As discussed recently regarding the six-country, Toy-Box study that found an effect of parenting on children’s intake of snack foods but not weight status, the consequences of what children eat for the development of overweight or obesity may be more pronounced as children age [48].

In its many forms, sugars and sodium make foods and beverages taste better because they can block/mask bad tastes [56]. That, along with the child’s heightened preferences for sweet and salty tastes [57], contributes to the enduring popularity of savory and sweet snacks, sweetened beverages, and syrups and other sugars, accounting for 18% of daily calories before the second birthday in this contemporary cohort of American toddlers. Consistent with research in older children [58], fruit accounted for less of toddlers’ energy intake than did snacks and sweets, and the more energy from snacks, sweetened beverages, and sweets at 1.5 years, the less energy from fruit and 100% fruit juices. The more added sugar in a child’s diet, the more likely the foods will be nutrient-poor [59] and displace healthier foods containing naturally occurring sugars, such as fruit [58]—important sources of cardio- and metabolic-protective nutrients. That children of this age are dependent on others to provide the foods they eat reinforces the importance of studying nutrition in the context of caregiver feeding practices and beliefs and in home and school environments [60,61].

The most recent 2020–2025 Dietary Guidelines for Americans recommended that children aged 2 years and younger should not be fed any food or beverages containing added sugar; after 2 years of age, Americans should limit added sugar intake to less than 10% of total daily energy needs [6]. The present data on a contemporary cohort of formula-fed infants in the United States revealed that added sugar intake already approached 10% of total daily energy needs by 1.5 years and that some children were already exceeding these guidelines by 1 year. Thus, interventions are needed to improve their diets well before children reach the age of 2 years [3].

## 5. Conclusions

In this follow-up study of children who participated in a longitudinal RCT, the formula induced differences in weight gain velocity during early infancy forecasted higher body weight status and the proportion of children with overweight during the second year [36,62] that were independent of the randomized treatment. The mechanisms underlying how transient rapid weight gain during infancy increases risk for later obesity [63] is an important area for future research [18,64,65,66]. Individual differences among mothers in how they structure their toddler’s food environment also predicted toddlers’ weight status. Tighter parental controls on snack foods predicted higher WLZ and overweight status at 1.5 years. As children vary in how they react to their environment [67], including how attracted they are to the taste of the foods they are fed, caregivers differ in when, what, and how they feed children [68,69], including their access to snack foods, which mothers may not view as “real foods” [43]. These data add to the pervasive body of evidence suggesting there are sensitive periods in early life during which rapid infancy weight gain and the food environment are modifiable determinants to prevent obesity and other diseases later in life [16,17,18,70].

## Figures and Tables

**Table 1:** Baseline characteristics (0.5 months) of 83 dyads who completed trial at 1 year.

Characteristic	Formula Treatment Group		*p*-Value ^1^
	CMF	EHF	
Number of dyads	44	39	
Infants			
Female	21 (48%)	20 (51%)	0.75
Race			0.15
Black	25 (57%)	22 (56%)	
White	14 (32%)	7 (18%)	
Other/more than one race	5 (11%)	10 (26%)	
Anthropometry, Z-scores			
Weight for age	−0.24 ± 0.13	−0.24 ± 0.13	1.00
Length for age	−0.37 ± 0.16	−0.43 ± 0.17	0.76
Weight for length	−0.23 ± 0.14	−0.14 ± 0.14	0.66
Mothers			
Age, years	27.9 ± 0.9	27.3 ± 0.9	0.66
Body Mass Index, kg/m^2^	31.6 ± 1.2	31.0 ± 1.3	0.73
Parity, primiparous	9 (20%)	8 (21%)	0.99
Household income			0.83
<$35,000	32 (73%)	26 (67%)	
$35,000–75,000	4 (9%)	4 (10%)	
>$75,000	8 (18%)	9 (23%)	
Education level			0.14
Primary school	6 (14%)	1 (3%)	
High school/technical school	26 (59%)	29 (74%)	
College degree or higher	12 (27%)	9 (23%)	
Data presented as *n* (%) or as mean ± standard error of mean (SEM). ^1^ *p*-values for main effect of infant formula treatment (CMF, cow milk formula, EHF, extensive protein hydrolysate formula) group.			

**Table 2:** Anthropometric outcomes by randomized infant formula treatment group of children who completed trial at 1 year.

Outcomes	Formula Treatment Group		*p*-Value ^1^
	CMF	EHF	
From 0.5 to 4.5 months	*n* = 44	*n* = 39	
Weight gain velocity (g/day)	29.2 ± 0.9	26.1 ± 1.0	0.02
Length gain velocity (cm/day)	0.102 ± 0.002	0.104 ± 0.002	0.62
From 4.5 months to 1 year			
Weight gain velocity (g/day)	12.5 ± 0.4	12.7 ± 0.5	0.71
Length gain velocity (cm/day)	0.050 ± 0.001	0.047 ± 0.001	0.14
At 1 year	*n* = 44	*n* = 39	
Weight for Length Z (WLZ)	0.78 ± 0.14	0.51 ± 0.17	0.20
At 1.5 years	*n* = 40	*n* = 38	
Weight for Length Z (WLZ)	0.80 ± 0.14	0.68 ± 0.17	0.57
Body weight status ^2^			0.10
Not overweight	20 (50%)	26 (68%)	
With overweight	20 (50%)	12 (32%)	
Data presented as mean ± standard error of mean (SEM) or as *n* (%). ^1^ *p*-values for main effect of infant formula treatment (CMF, cow milk formula; EHF, extensive protein hydrolysate) group for the children who remained in the trial until 1 year (*n* = 83) and then 1.5 years (*n* = 78). ^2^ Categories based on age- and sex-specific WLZ percentiles, which defines overweight as values greater than the 85th percentile (WLZ > 1.0364).			

**Table 3:** Mean daily intake and percent (%) daily energy by food category at 1 and 1.5 years.

Category	Age of Toddlers		*p*-Value ^1^
	1 Year	1.5 Years	
Total daily intake			
Energy, kcal/day	1169 ± 49	1367 ± 57	<0.001
Sodium, mg/day	1411 ± 74	2209 ± 120	<0.001
Added sugar ^2^, kcal/day	64 ± 8	129 ± 13	<0.001
WWEIA food categories ^3^, kcal/day			
Infant formula	399 ± 42	9 ± 9	<0.001
Snacks and sweets	67 ± 12	183 ± 27	<0.001
Sweetened beverages	21 ± 5	77 ± 12	<0.001
Sugars	9 ± 3	6 ± 12	0.34
Fruit	45 ± 6	66 ± 2	0.02
100% fruit juice	39 ± 9	58 ± 8	0.07
Percent daily energy, %			
Added sugar	5 ± 1	9 ± 15	<0.001
WWEIA food categories ^2^			
Infant formula	33 ± 3	1 ± 1	<0.001
Snacks and sweets	5 ± 1	12 ± 1	<0.001
Sweetened beverages	2 ± 1	5 ± 1	<0.001
Sugars	0.7 ± 0.2	0.4 ± 0.2	0.28
Fruit	4 ± 1	5 ± 1	0.30
100% fruit juice	3 ± 1	4 ± 1	0.15
Data presented as mean ± standard error of mean (SEM). ^1^ *p*-values for main effect of age; t-test for dependent samples. Number enrolled was *n* = 83 at 1 year and *n* = 78 at 1.5 years. Diet records were available for *n* = 67 toddlers at 1 year and *n* = 73 at 1.5 years. ^2^ Labeling requirement for “added sugars” is not required for infant formula, which has specific labeling regulations in 21 CFR 107.100 [32]. ^3^ Selected food categories from What We Eat in America (WWEIA) [29]. Snacks and sweets category includes baby-food snacks and sweets and fruit category includes baby-food fruit.			

**Table 4:** Generalized linear mixed models on trajectory of WLZ score from 1 year to 1.5 years.

Model	Coefficient ± SEM	*p*-Value
Weight gain velocity, 0.5–4 months	0.09 ± 0.02	<0.001
Allow Access ^1^	−0.25 ± 0.27	0.35
Visit	0.67 ± 0.15	<0.001
Formula treatment group ^2^	−0.09 ± 1.30	0.95
Weight gain velocity, 0.5–4 months × visit	−0.01 ± 0.00	<0.001
Allow Access × visit	−0.09 ± 0.04	<0.001
Formula treatment group × visit	−0.01 ± 0.04	0.83
Weight gain velocity × formula treatment group	0.01 ± 0.03	0.64
Allow Access × formula treatment group	−0.08 ± 0.33	0.82
^1^ The Allow Access construct of the Toddler Snack Food Feeding Questionnaire measures how much mothers allow their children access to sweets and snacks [20]. ^2^ Reference group, CMF (cow milk formula) treatment group.		

## Data Availability

Data described in the manuscript and codebook will be made available upon request pending application.

## References

[1] Dattilo A.M., Birch L., Krebs N.F., Lake A., Taveras E.M., Saavedra J.M. (2012). Need for early interventions in the prevention of pediatric overweight: A review and upcoming directions. J. Obes..

[2] Mennella J.A., Forestell C.A., Ventura A.K., Fisher J.O., Lockman J.J., Tamis-LeMonda C.S. (2020). The development of infant feeding. The Cambridge Handbook of Infant Development.

[3] Raiten D.J., Raghavan R., Porter A., Obbagy J.E., Spahn J.M. (2014). Executive summary: Evaluating the evidence base to support the inclusion of infants and children from birth to 24 mo of age in the Dietary Guidelines for Americans—“The B-24 Project”. Am. J. Clin. Nutr..

[4] Robinson S.M. (2015). Infant nutrition and lifelong health: Current perspectives and future challenges. J. Dev. Orig. Health Dis..

[5] Pérez-Escamilla R., Segura-Pérez S., Moran V.H. (2019). Dietary guidelines for children under 2 years of age in the context of nurturing care. Matern. Child Nutr..

[6] US Department of Agriculture and US Department of Health and Human Services 2020–2025 Dietary Guidelines for Americans, 9th Edition. https://www.dietaryguidelines.gov.

[7] Koletzko B., Hirsch N.L., Jewell J.M., Dos Santos Q., Breda J., Fewtrell M., Weber M.W. (2020). National recommendations for infant and young child feeding in the World Health Organization European region. J. Pediatr. Gastroenterol. Nutr..

[8] Dewey K.G., Pannucci T., Casavale K.O., Davis T.A., Donovan S.M., Kleinman R.E., Taveras E.M., Bailey R.L., Novotny R., Schneeman B.O. (2021). Development of food pattern recommendations for infants and toddlers 6–24 months of age to support the Dietary Guidelines for Americans, 2020–2025. J. Nutr..

[9] Dewey K.G. (1998). Growth characteristics of breast-fed compared to formula-fed infants. Biol. Neonate.

[10] Armstrong J., Reilly J.J. (2002). Breastfeeding and lowering the risk of childhood obesity. Lancet.

[11] Grummer-Strawn L.M., Mei Z. (2004). Does breastfeeding protect against pediatric overweight? Analysis of longitudinal data from the Centers for Disease Control and Prevention Pediatric Nutrition Surveillance System. Pediatrics.

[12] Owen C.G., Martin R.M., Whincup P.H., Smith G.D., Cook D.G. (2005). Effect of infant feeding on the risk of obesity across the life course: A quantitative review of published evidence. Pediatrics.

[13] Mennella J.A., Inamdar L., Pressman N., Schall J.I., Papas M.A., Schoeller D., Stallings V.A., Trabulsi J.C. (2018). Type of infant formula increases early weight gain and impacts energy balance: A randomized controlled trial. Am. J. Clin. Nutr..

[14] Rzehak P., Sausenthaler S., Koletzko S., Reinhardt D., Von Berg A., Krämer U., Berdel D., Bollrath C., Grübl A., German Infant Nutritional Intervention Study Group (2009). Short- and long-term effects of feeding hydrolyzed protein infant formulas on growth at ≤6 y of age: Results from the German Infant Nutritional Intervention Study. Am. J. Clin. Nutr..

[15] Mennella J.A., Ventura A.K., Beauchamp G.K. (2011). Differential growth patterns among healthy infants fed protein hydrolysate or cow-milk formulas. Pediatrics.

[16] Monteiro P.O.A., Victora C. (2005). Rapid growth in infancy and childhood and obesity in later life—A systematic review. Obes. Rev..

[17] Woo Baidal J.A., Locks L.M., Cheng E.R., Blake-Lamb T.L., Perkins M.E., Taveras E.M. (2016). Risk factors for childhood obesity in the first 1,000 days: A systematic review. Am. J. Prev. Med..

[18] Zheng M., Lamb K., Grimes C., Laws R., Bolton K., Ong K.K., Campbell K. (2018). Rapid weight gain during infancy and subsequent adiposity: A systematic review and meta-analysis of evidence. Obes. Rev..

[19] Rossen L.M., Simon A.E., Herrick K.A. (2016). Types of infant formulas consumed in the United States. Clin. Pediatr..

[20] Corsini N., Wilson C., Kettler L., Danthiir V. (2010). Development and preliminary validation of the Toddler Snack Food Feeding Questionnaire. Appetite.

[21] Blaine R.E., Kachurak A., Davison K.K., Klabunde R., Fisher J.O. (2017). Food parenting and child snacking: A systematic review. Int. J. Behav. Nutr. Phys. Act..

[22] Corsini N., Kettler L., Danthiir V., Wilson C. (2018). Parental feeding practices to manage snack food intake: Associations with energy intake regulation in young children. Appetite.

[23] Rzehak P., Sausenthaler S., Koletzko S., Reinhardt D., Von Berg A., Krämer U., Berdel D., Bollrath C., Grübl A., Bauer C.P. (2011). Long-term effects of hydrolyzed protein infant formulas on growth—Extended follow-up to 10 y of age: Results from the German Infant Nutritional Intervention (GINI) study. Am. J. Clin. Nutr..

[24] Pew Charitable Trusts Philadelphia 2021: The State of the City. https://www.pewtrusts.org/en/research-and-analysis/reports/2021/04/philadelphia-2021-state-of-the-city.

[25] WHO Multicentre Growth Reference Study Group (2006). World Health Organization Child. Growth Standards: Length/Height-for-Age, Weight-for-Age, Weight-for-Length, Weight-for-Height and Body Mass Index-for-Age: Methods and Development.

[26] Butte N.F., Wong W.W., Hopkinson J.M., Smith E.O., Ellis K.J. (2000). Infant Feeding Mode Affects Early Growth and Body Composition. Pediatrics.

[27] Roy S.M., Spivack J.G., Faith M.S., Chesi A., Mitchell J.A., Kelly A., Grant S.F., McCormack S.E., Zemel B.S. (2016). Infant BMI or weight-for-length and obesity risk in early childhood. Pediatrics.

[28] Wood C.T., Skinner A.C., Yin H.S., Rothman R.L., Sanders L.M., Delamater A.M., Perrin E.M. (2016). Bottle size and weight gain in formula-fed infants. Pediatrics.

[29] United States Department of Agriculture, Agricultural Research Service What We Eat in America Food Categories 2017–2018. https://www.ars.usda.gov/northeast-area/beltsville-md-bhnrc/beltsville-human-nutrition-research-center/food-surveys-research-group/docs/wweianhanes-overview/.

[30] Herrick K.A., Fryar C.D., Hamner H.C., Park S., Ogden C.L. (2020). Added Sugars Intake among US Infants and Toddlers. J. Acad. Nutr. Diet..

[31] Added Sugars on the New Nutrition Facts Label. https://www.fda.gov/food/new-nutrition-facts-label/added-sugars-new-nutrition-facts-label.

[32] US Department of Health & Human Services, Food and Drug Administration, Center for Food Safety and Applied Nutrition Labeling of Infant Formula: Guidance for Industry. https://www.fda.gov/regulatory-information/search-fda-guidance-documents/guidance-industry-labeling-infant-formula.

[33] Catalano P.M., Farrell K., Thomas A., Huston-Presley L., Mencin P., De Mouzon S.H., Amini S.B. (2009). Perinatal risk factors for childhood obesity and metabolic dysregulation. Am. J. Clin. Nutr..

[34] Stunkard A.J., Berkowitz R.I., Stallings V.A., Schoeller D.A. (1999). Energy intake, not energy output, is a determinant of body size in infants. Am. J. Clin. Nutr..

[35] Taveras E.M., Rifas-Shiman S.L., Belfort M.B., Kleinman K.P., Oken E., Gillman M.W. (2009). Weight status in the first 6 months of life and obesity at 3 years of age. Pediatrics.

[36] Mennella J.A., Reiter A., Brewer B., Pohlig R.T., Stallings V.A., Trabulsi J.C. (2020). Early weight gain forecasts accelerated eruption of deciduous teeth and later overweight status during the first year. J. Pediatr..

[37] Deming D.M., Reidy K.C., Fox M.K., Briefel R.R., Jacquier E., Eldridge A.L. (2017). Cross-sectional analysis of eating patterns and snacking in the US Feeding Infants and Toddlers Study 2008. Public Health Nutr..

[38] Ziegler P., Hanson C., Ponza M., Novak T., Hendricks K. (2006). Feeding Infants and Toddlers Study: Meal and snack intakes of Hispanic and non-Hispanic infants and toddlers. J. Am. Diet. Assoc..

[39] Fisher J.O., Birch L.L. (2000). Parents’ restrictive feeding practices are associated with young girls’ negative self-evaluation of eating. J. Am. Diet. Assoc..

[40] Beckers D., Karssen L.T., Vink J.M., Burk W.J., Larsen J.K. (2021). Food parenting practices and children’s weight outcomes: A systematic review of prospective studies. Appetite.

[41] Burrows T.L., Martin R.J., Collins C.E. (2010). A systematic review of the validity of dietary assessment methods in children when compared with the method of doubly labeled water. J. Am. Diet. Assoc..

[42] Fisher J.O., Butte N.F., Mendoza P.M., Wilson T.A., Hodges E.A., Reidy K.C., Deming D. (2008). Overestimation of infant and toddler energy intake by 24-h recall compared with weighed food records. Am. J. Clin. Nutr..

[43] Fisher J., Wright G., Herman A., Malhotra K., Serrano E., Foster G., Whitaker R. (2015). “Snacks are not food”. Low-income, urban mothers’ perceptions of feeding snacks to their preschool-aged children. Appetite.

[44] Hess J.M., Jonnalagadda S.S., Slavin J.L. (2016). What is a snack, why do we snack, and how can we choose better snacks? A review of the definitions of snacking, motivations to snack, contributions to dietary intake, and recommendations for improvement. Adv. Nutr..

[45] Johnson G.H., Anderson G.H. (2010). Snacking definitions: Impact on interpretation of the literature and dietary recommendations. Crit. Rev. Food Sci. Nutr..

[46] Younginer N.A., Blake C.E., Davison K.K., Blaine R.E., Ganter C., Orloski A., Fisher J.O. (2016). “What do you think of when I say the word ‘snack’?” Towards a cohesive definition among low-income caregivers of preschool-age children. Appetite.

[47] Blaine R.E., Fisher J.O., Taveras E.M., Geller A.C., Rimm E.B., Land T., Perkins M., Davison K.K. (2015). Reasons low-income parents offer snacks to children: How feeding rationale influences snack frequency and adherence to dietary recommendations. Nutrients.

[48] Gibson E.L., Androutsos O., Moreno L., Flores-Barrantes P., Socha P., Iotova V., Cardon G., De Bourdeaudhuij I., Koletzko B., Skripkauskaite S. (2020). Influences of parental snacking-related attitudes, behaviours and nutritional knowledge on young children’s healthy and unhealthy snacking: The ToyBox study. Nutrients.

[49] Bellisle F. (2014). Meals and snacking, diet quality and energy balance. Physiol. Behav..

[50] Duffy E.W., Kay M.C., Jacquier E., Catellier D., Hampton J., Anater A.S., Story M. (2019). Trends in food consumption patterns of US infants and toddlers from Feeding Infants and Toddlers Studies (FITS) in 2002, 2008, 2016. Nutrients.

[51] Shriver L.H., Marriage B., Bloch T.D., Spees C.K., Ramsay S.A., Taylor C.A. (2015). Nutritional composition and the contribution of snacks to the dietary intakes of 2–5 year old children. J. Acad. Nutr. Diet..

[52] Liu J., Lee Y., Micha R., Li Y., Mozaffarian D. (2021). Trends in junk food consumption among US children and adults, 2001–2018. Am. J. Clin. Nutr..

[53] Vos M.B., Kaar J.L., Welsh J.A., Van Horn L.V., Feig D.I., Anderson C.A.M., Patel M.J., Munos J.C., Krebs N.F., Xanthakos S.A. (2016). Added sugars and cardiovascular disease risk in children: A scientific statement from the American Heart Association. Circulation.

[54] Roess A.A., Jacquier E.F., Catellier D.J., Carvalho R., Lutes A.C., Anater A.S., Dietz W.H. (2018). Food consumption patterns of infants and toddlers: Findings from the Feeding Infants and Toddlers Study (FITS) 2016. J. Nutr..

[55] Maalouf J., Cogswell M.E., Yuan K., Martin C., Gunn J.P., Pehrsson P., Merritt R., Bowman B. (2015). Top sources of dietary sodium from birth to age 24 mo, United States, 2003–2010. Am. J. Clin. Nutr..

[56] Mennella J.A., Reed D.R., Roberts K.M., Mathew P.S., Mansfield C.J. (2014). Age-related differences in bitter taste and efficacy of bitter blockers. PLoS ONE.

[57] Mennella J.A., Finkbeiner S., Lipchock S.V., Hwang L.D., Reed D.R. (2014). Preferences for salty and sweet tastes are elevated and related to each other during childhood. PLoS ONE.

[58] Drewnowski A., Rehm C. (2014). Consumption of added sugars among US children and adults by food purchase location and food source. Am. J. Clin. Nutr..

[59] Alexy U., Sichert-Hellert W., Kersting M. (2003). Associations between intake of added sugars and intakes of nutrients and food groups in the diets of German children and adolescents. Br. J. Nutr..

[60] Meleleo D., Susca G., Andrulli Buccheri V., Lamanna G., Cassano L., De Chirico V., Mustica S., Caroli M., Bartolomeo N. (2021). Effectiveness of an innovative sensory approach to improve children’s nutritional choices. Int. J. Environ. Res. Public Health.

[61] Larsen J.K., Hermans R.C., Sleddens E.F., Engels R.C., Fisher J.O., Kremers S.P. (2015). How parental dietary behavior and food parenting practices affect children’s dietary behavior. Interacting sources of influence?. Appetite.

[62] Trabulsi J.C., Smethers A.D., Eosso J.R., Papas M.A., Stallings V.A., Mennella J.A. (2020). Impact of early rapid weight gain on odds for overweight at one year differs between breastfed and formula-fed infants. Pediatr. Obes..

[63] Baird J., Fisher D., Lucas P., Kleijnen J., Roberts H., Law C. (2005). Being big or growing fast: Systematic review of size and growth in infancy and later obesity. BMJ.

[64] Ong K.K., Emmett P., Northstone K., Golding J., Rogers I., Ness A.R., Wells J.C., Dunger D.B. (2009). Infancy weight gain predicts childhood body fat and age at menarche in girls. J. Clin. Endocrinol. Metab..

[65] Salgin B., Norris S.A., Prentice P., Pettifor J., Richter L.M., Ong K.K., Dunger P.D. (2015). Even transient rapid infancy weight gain is associated with higher BMI in young adults and earlier menarche. Int. J. Obes..

[66] Loo E.X.-L., Goh A., Aris I.B.M., Teoh O.H., Shek L.P.-C., Lee B.W., Chan Y.H., Tint M.T., Soh S.-E., Saw S.-M. (2017). Effects of infant weight gain on subsequent allergic outcomes in the first 3 years of life. BMC Pediatr..

[67] Rothbart M.K., Ellis L.K., Posner M.I., Baumeister R.F., Vohs K.D. (2011). Temperament and self-regulation. Handbook of Self-Regulation: Research, Theory and Applications.

[68] Spill M.K., Callahan E.H., Shapiro M.J., Spahn J.M., Wong Y.P., Benjamin-Neelon S.E., Birch L., Black M.M., Cook J.T., Faith M.S. (2019). Caregiver feeding practices and child weight outcomes: A systematic review. Am. J. Clin. Nutr..

[69] Davison K.K., Blake C.E., Blaine R.E., Younginer N.A., Orloski A., Hamtil H.A., Ganter C., Bruton Y.P., Vaughn A.E., Fisher J.O. (2015). Parenting around child snacking: Development of a theoretically-guided, empirically informed conceptual model. Int. J. Behav. Nutr. Phys. Act..

[70] Trabulsi J.C., Mennella J.A. (2012). Diet, sensitive periods in flavour learning, and growth. Int. Rev. Psychiatry.

